# Low Protein Intakes and Poor Diet Quality Associate with Functional Limitations in US Adults with Diabetes: A 2005–2016 NHANES Analysis

**DOI:** 10.3390/nu13082582

**Published:** 2021-07-27

**Authors:** Stephanie M. Fanelli, Owen J. Kelly, Jessica L. Krok-Schoen, Christopher A. Taylor

**Affiliations:** 1 Medical Dietetics, The Ohio State University, Columbus, OH 43210, USA; fanelli.18@buckeyemail.osu.edu; 2 Department of Molecular and Cellular Biology, Sam Houston State University, Conroe, TX 77304, USA; ojk003@shsu.edu; 3 School of Health and Rehabilitation Sciences, The Ohio State University, Columbus, OH 43210, USA; jessica.schoen@osumc.edu

**Keywords:** diabetes, protein, diet quality, dietary patterns, NHANES, sarcopenia, physical limitations

## Abstract

Type 2 diabetes is associated with an increased risk for sarcopenia. Moreover, sarcopenia correlates with increased risk for falls, fractures, and mortality. This study aimed to explore relationships among nutrient intakes, diet quality, and functional limitations in a sample of adults across levels of glycemic control. Data were examined from 23,487 non-institutionalized adults, 31 years and older, from the 2005–2016 National Health and Nutrition Examination Survey. Hemoglobin A1c (%) was used to classify level of glycemic control: non-diabetes (<5.7%); pre-diabetes (5.7–6.4%); diabetes (≥6.5%). Dietary data were collected from a single 24-h dietary recall. Participants were categorized as meeting or below the protein recommendation of 0.8 g/kg of body weight. Physical functioning was assessed across 19-discrete physical tasks. Adults below the protein recommendation consumed significantly more carbohydrate and had lower diet quality across all glycemic groups compared to those who met the protein recommendation (*p* < 0.001). Adults with diabetes who did not meet protein recommendations had significantly poorer diet quality and significantly higher mean number of functional limitations. A greater percent of adults with diabetes who did not meet the protein recommendation reported being physically limited for most activities, with more than half (52%) reporting limitations for stooping, crouching, and kneeling. This study underscores the potential for physical limitations associated with low protein intakes, especially in adults with diabetes. In the longer term, low protein intakes may result in increased risk of muscle loss, as protein intake is a critical nutritional factor for prevention of sarcopenia, functional limitations, and falls.

## 1. Introduction

Diet plays a vital role in maintaining health and preventing and managing chronic disease. Type 2 diabetes (diabetes) is a progressive disease affecting more than 30 million adults in the US [1]. Prevalence is expected to increase due to rising rates of obesity, physical inactivity, aging, and urbanization [2,3]. In addition to increased morbidity and mortality risk [4], diabetes is associated with sarcopenia, a condition of low muscle mass, function, and quality, further linked to physiological aging [5,6], and impaired physical functioning [7,8]. Sarcopenia is associated with increased risk for falls and fractures in individuals with diabetes [9], as well as decreased quality of life and frailty [10]. Hyperglycemia and insulin resistance may be central to the development of sarcopenia in aging and may also be related to time from diabetes diagnosis [11] due to micro- and macrovascular complications [12]. Although age remains the strongest risk factor [13], hyperglycemia and insulin resistance have been shown to be independent risk factors for sarcopenia [7].

In addition to physical activity, nutrition interventions, particularly addressing protein intakes, may help prevent and manage sarcopenia [14,15]. The management of sarcopenia is particularly important for older adults as they are at increased risk of falls and fractures. However, research has indicated deficiency in the protein intake of older adults and call for increasing the Recommended Dietary Allowance (RDA) for protein for older adults [16,17,18]. Additionally, oral medications that are commonly prescribed in mid-life and older adult populations, such as statins, sulfonylureas and glinides, may adversely affect muscle function [19]. Therefore, adequate protein intakes and healthy overall dietary quality [20] are important in the prevention and management of sarcopenia and to maintain functionality. With the evidence lacking for the role of diet and protein intakes in sarcopenia for individuals with diabetes, this study aimed to identify if differences in dietary protein intakes and diet quality are associated with functional limitations in adults with different levels of glycemic control. Examining protein intake and diet quality may provide insight into the relationships between nutrition and functional outcomes in the U.S. population with and without diabetes.

## 2. Materials and Methods

### 2.1. Sample Population

Data were examined from 23,487 non-institutionalized adults aged 31 years and older from the 2005–2016 National Health and Nutrition Examination Survey (NHANES). NHANES is a national nutrition monitoring system that utilized a complex, multistage sampling design to select participants to create a nationally representative sample [21]. Participants with a completed dietary recall interview, measured body weight and glycated hemoglobin data were included in the analyses. To address standard protein recommendations per kilogram of body, women who were pregnant were excluded from the analysis. Demographic and personal data were gathered during in-home interviews, which included gender, race, ethnicity, marital status, education, and income level as a percent of the federal poverty rate. The National Center for Health Statistics Research Ethics Review Board approved all methods carried out in the series of surveys [22].

### 2.2. Glycemic Measures

Glycated hemoglobin (A1c%) was used to categorize levels of glycemia and indicate the degree of blood glucose control from the prior 90 days. These data were examined from blood collected during the visit to the Mobile Examination Center. A1c data were used to classify sample participants into glycemic groups: nondiabetes (A1c < 5.7%), prediabetes (A1c 5.7–6.4%), and diabetes (A1c ≥ 6.5%) using criteria from the American Diabetes Association [23].

### 2.3. Dietary Intakes and Diet Quality

Estimates of dietary intake data were assessed from the 24-h dietary recall, which utilized the Automated Multiple Pass Method conducted by trained interviewers in the Mobile Examination Center [24]. This multi-step method allowed for improved accuracy of the intakes of foods and beverages reported as consumed from midnight to midnight of the prior day [25,26]. Nutrient and MyPlate intakes were estimated from the Food and Nutrient Database for Dietary Studies as well as the Food Patterns Equivalents Database, respectively, to estimate intakes on the day of record. For a better comparison of nutrient density across varying levels of energy intakes, energy-adjusted nutrient estimations were made represent intakes per 1000 kcals. Meal consumption and skipping were assessed based on the presence or absence of foods or beverages during self-classification of the meal occasion (breakfast, lunch, dinner, snack) during dietary recalls.

Diet quality was assessed using the Healthy Eating Index (HEI)-2015, which measures the alignment of dietary intakes with the 2015–2020 Dietary Guidelines for Americans (DGA) [27]. The HEI-2015 features 13 graded components and operates on a 100-point scale for maximum score. This density-based scale measures amounts per 1000 kcal, with higher scores on each of the subscales and the total score represent better diet quality related to the DGA. For a metric of comparison of nutrient intakes to reference standards to identify potential gaps, the percent of participants meeting the Estimated Average Requirement (EAR) or Adequate Intake (AI) for select nutrients were classified from the day of intake.

### 2.4. Protein Intake Recommendations

To assess dietary differences for those who were below protein intake recommendations on the day of intake, protein intake estimations and measured body weights were used to stratify each participant as either meeting or being below the protein recommendation of 0.8 g of dietary protein per kilogram of body weight per day (0.8 g/kg/day) [28].

### 2.5. Physical Functioning

Data from the physical functioning questionnaire were used to identify self-reported physical limitations for 19 discrete physical tasks. The questionnaire was conducted by trained interviewers in participants’ homes and utilized the Computer-Assisted Personal Interview system. The numbers of physical limitations reported was calculated as the sum of physical activities that was reported as a limitation for each individual. Combined isometric grip strength (kg) was calculated by averaging all dynamometer results for each participant (3 measurements per hand) to assess a direct function strength measure.

### 2.6. Statistical Analyses

The 2005–2016 NHANES data were gathered from publicly available sources and imported into SPSS (version 25, IBM SPSS Inc., Chicago, IL, USA). Weighted population-based estimates were calculated using sample weights, clusters, and strata provided by the Centers for Disease Control and Prevention’s National Center for Health Statistics. Analyses were conducted with SPSS Complex Samples to generate nationally representative estimates.

This cross-sectional analysis categorized adults aged 31 years and older into levels of glycemic control (non-diabetes, pre-diabetes, and diabetes) and then further classified by whether recommended protein intakes were met (intake ≥ 0.8 g/kg/day) or not met (intake < 0.8 g/kg/day). Analysis of covariance was performed to test for differences in energy and nutrient intakes, diet quality, and the number of limitations per group across glycemic and protein groups, controlled for sex, race, ethnicity, marital status, and percent of federal poverty rate. Frequency analyses were used to identify weighted population percents of adults meeting DRI reference intakes and those experiencing physical limitations per protein and glycemic stratification. To account for multiple comparisons, significance was set a priori at α ≤ 0.01.

## 3. Results

### 3.1. Demographics

Data from 23,487 adults aged 31 years and older were stratified into non-diabetes (*n* = 14,730), pre-diabetes (*n* = 5869), and diabetes (*n* = 2888) based on A1c values and further categorized into protein intake groups (not met < 0.8 g/kg/day: *n* = 9514; met ≥ 0.8 g/kg/day: *n* = 13,973; Table 1). Overall, 51.2% of those with diabetes, 45.4% of those with pre-diabetes and 34.2% of those without diabetes did not meet the individual protein recommendation. Across all glycemic groups, more males (73% of non-diabetes, 62% of pre-diabetes, and 57% of diabetes) met the protein intake recommendations compared to females (60% of non-diabetes, 49% of pre-diabetes, and 39% of diabetes). A greater proportion of participants did not meet protein intake recommendations for both sexes in the diabetes group (43% of males and 61% of females) compared to the non-diabetes (27% of males and 40% of females) and pre-diabetes groups (38% of males and 52% of females). The mean BMIs for participants who did not meet the protein recommendations was higher across all groups than that of adults who met protein recommendations. Non-Hispanic blacks and participants who were single, divorced, or widowed were least likely to meet protein recommendations across all glycemic groups.

### 3.2. Dietary Intakes

For those who met or exceeded the protein intake recommendation on the day of intake, the mean total protein intake was double that of those who did not meet the recommendation (Table 2). Energy-adjusted protein intakes was greater for all groups across glycemic status, with adults with diabetes having the greatest density (46 g protein per 1000 kcal). Participants who did not meet protein recommendations consumed significantly less energy for all glycemic groups; 66%, 71% and 65% of the energy intakes in the non-diabetes, pre-diabetes, and diabetes groups, respectively (*p* < 0.001). Additionally, adults not meeting protein recommendations consumed significantly more total carbohydrate and added sugars across all glycemic groups (*p* < 0.001). Across all glycemic groups, adults below the protein intake recommendation consumed significantly less total fat, saturated fat, monounsaturated fat, choline, vitamin B12, phosphorus, zinc, sodium, and selenium per 1000 kcal (*p* ≤ 0.007).

Many adults below the recommended protein intake did not meet Estimated Average Requirements (EAR) or Adequate Intakes (AI) for fiber (≥93%), magnesium (≥82%), choline (≥96%), and vitamins C (≥65%), D (≥95%), E (≥92%), and K (≥78%) across all glycemic groups (Table 3). Even for those meeting protein recommendations, many participants across glycemic groups did not meet the EAR/AI for fiber (≥ 79%), vitamin C (≥50%), choline (≥72%), and vitamins D (≥84%), E (≥74%), and K (≥62%). Rates of inadequate intakes were lower from the below protein groups compared to the met protein groups for B vitamins, including niacin, thiamin, riboflavin, vitamin B6 and B12, as well as iron, zinc, magnesium, copper, and selenium.

### 3.3. Diet Quality

Across all glycemic groups, total diet quality was poorer in adults below the protein intake recommendation (*p* < 0.001; Table 4). HEI scores were less than 70% of the ideal score for greens and beans, whole grains, dairy, seafood and plant proteins, fatty acids, refined grains, sodium, and saturated fat. Those that met the protein intake recommendation had significantly poorer HEI scores for sodium and saturated fat across all glycemic levels (*p* < 0.001). Adults with diabetes who met protein recommendations had better HEI-2015 scores for total vegetables, whole grains, dairy, and added sugars than any other group, and in contrast, they had the poorest HEI-2015 score for sodium.

### 3.4. Physical Functioning Limitations

Adults with diabetes had greater frequency of physical limitations than those with non-diabetes, as did adults with pre-diabetes, regardless of protein intake category (Table 5). Those not meeting protein recommendations reported significantly more numbers of physical limitations than those who met protein recommendation across all levels of glycemia. Physical limitations were more prevalent in adults with protein intakes below 0.8 g/kg/day compared to those who met (or exceeded) this recommendation across all glycemic groups. The most common limitations across all groups included: stooping, crouching, and kneeling; standing for long periods; and pushing or pulling large objects. More than half (52%) of the diabetes population that was below protein recommendations reported physical limitations for stooping, crouching, and kneeling.

Grip strength and number of physical limitations were analyzed for both adjusted and unadjusted results. Unadjusted data reveal greater grip strength among individuals who met protein recommendations compared to those below protein recommendations across all glycemic groups. Adults in the non-diabetes group had the greatest overall grip strength compared to those with pre-diabetes and diabetes. After controlling for factors related to diet quality and diabetes, mean grip strength was lowest in the diabetes groups.

## 4. Discussion

Diet quality has been previously examined in the general US population, yet less is known regarding diet quality in the adult population with diabetes [29,30,31,32,33]. Moreover, key components of diet quality, such as protein, likely contribute to physical functioning limitations in this population [18]. Therefore, this study aimed to identify differences in diet quality, nutrient intakes, and physical limitations by protein intake across levels of glycemic control. The current analysis found that adults with protein intakes not meeting the recommendation have poor overall diet quality and greater physical limitations regardless of diabetes status.

To optimize personal functioning and health outcomes from diabetes, medical nutrition therapy (MNT) is included in diabetes care guidelines as a frontline therapy and is recommended for all individuals with prediabetes and diabetes [34,35]. Although nutrition therapy aims to target overall diet quality, it tends to focus nutrition education related to carbohydrate and added sugars intakes, to improve glycemic control [36,37,38]. While emphasizing the importance of regulating carbohydrate intake and reducing calories is beneficial in this population, it may take attention away from other key components of diet quality, such as fruits, vegetables, and protein. In our analysis adults with diabetes who met protein recommendations had better overall HEI-2015 scores than any other group for total vegetables, whole grains, dairy, and added sugars, highlighting the impact of nutrition education focused on carbohydrate and added sugars. However, our results also indicate that protein choices among adults with diabetes are likely less lean options, consisting of fatty and processed meats, resulting in greater consumption of saturated fat and a reduction in diet quality. This underscores the limitations of current nutrition education with respect to the importance of protein intakes, specifically lean and plant-based proteins, especially for cost effective sources for intakes among individuals in a lower socioeconomic status. Considering that adults with diabetes are already at an increased risk for cardiovascular disease, it is important that they choose leaner protein options to decrease saturated fat intake and improve serum lipids and meet protein needs [39,40].

Dietary protein can help with glycemic control as it is insulinotropic [41]. It may help slow gastric emptying, improve glucagon-like peptide 1 (GLP-1) secretion [42], and increase satiety [43]. Additionally, consumption of lean protein may help to improve post-prandial dysmetabolism rather than contribute to blood glucose exacerbations [44]. Our analysis found that adults who met protein intakes generally reported eating less total carbohydrates, whereas adults not meeting these modest protein recommendations relied more heavily on carbohydrates and sugars.

The current study identified that adults who did not meet protein recommendations had poorer nutrient density, overall diet quality, and were more likely to skip meals. These factors likely contribute to greater rates of physical limitations in this population [18]. The number of physical limitations was greatest in adults with diabetes and is likely due to insulin resistance as well as other metabolic derangements [7,10]. Poorer diet quality, and lower intakes of key nutrients, are also likely to negatively impact physical functioning [45] and loss of lean body mass [46].

Typically, the incidence of diabetes is higher in the older population; however, rates are increasing in adults younger than 65 years [1]. While age was not used as a stratification but as a covariate in this analysis, previous findings showed that older adults with poorer protein intakes are more likely to skip meals and have greater rates of physical limitations than those of younger ages [18]. Missing meals creates a lost opportunity for obtaining energy and key nutrients to support normal health and physical function. That analysis was stratified by age only and did include people with diabetes. The compounding accumulation of age, poor diet quality, lower protein intake, and lower energy intake are likely responsible for the greater rates of physical limitations. This reduction in physical function may be considered a major risk factor for sarcopenia [47].

Previous studies have identified associations between sarcopenia and diabetes [5,48,49]. Although the exact mechanism is unclear, insulin resistance is associated with loss of muscle tissue even in adults without a diabetes diagnosis [50]. Therefore, while loss of function and sarcopenia continue to be health concerns for older adults, it may be more important for those with diabetes. Sarcopenia is a health concern as it may increase the risk for falls, and subsequently fractures, as well as decreased physical functioning [9] and evidence suggests that adults with diabetes have an increased risk for falls and fractures [51]. Considering the milieu of increased risks for acute and chronic health events in diabetes, a greater emphasis on diet quality and adequate protein is needed.

Prior data has suggested that, at the population level, adults may be consuming sufficient protein, especially amongst males [52]; however, at the individual level, nearly half of adults with diabetes did not meet the recommended protein level on the day of intake. Several groups have encouraged recommendations for higher recommended levels for protein intakes (1.0 or 1.2 g per kilogram) [53,54,55,56]; thus, the proportion of adults not meeting recommended protein intakes on the day of intakes would be greater. These lower protein intakes, especially in adults with diabetes, were associated with poorer diet quality, not meeting nutrient recommendations, and increased numbers of physical limitations compared to those who met or exceeded the protein recommendation. These findings add to the body of evidence that sufficient protein intakes may support functional outcomes while increasing the emphasis on overall diet quality.

These data provide a unique opportunity to assess function status related to dietary intakes in individuals by glycemic level. However, there are limitations to consider when evaluating the data. The dietary intakes represent a single, 24-h recall and cannot be assumed to represent usual intakes do not establish nutrient adequacy and are subject to recall bias. The multiple pass method employed in data collection helps improve the accuracy of the reporting [24]. Furthermore, as a cross-sectional analyses, these data do not infer causation of diabetes status, dietary patterns, and physical functioning outcomes, but underscore differences across the groups on the day of intake.

Based on the day of intake, these findings challenge the assumption that all Americans, and especially those with diabetes, are getting enough protein [57,58]. Those that did not meet protein intakes on the day of intake had lower energy intakes, but also significantly lower protein intakes per 1000 kcals. Assessment on physical function and dietary proteins could be a target of assessment, especially in individuals with comorbidities such as diabetes. These concerns of physical functioning limitations have been seen in as early as middle-aged adults, suggesting screening should not be limited just to the elderly [18]. MNT should emphasize improvement of overall diet quality, including adequate protein intake from a variety of sources. However, due to the frequency of meal skipping, likely related to high professional, social, and familial responsibilities, food intake alone may be insufficient to meet protein and micronutrient needs, while not exceeding energy needs [18,59]. Therefore, when dietary intakes may not be sufficient to meet dietary needs for varying reasons, integrating diabetes-specific oral nutrition supplements with regular food intake would increase the likelihood of meeting protein and micronutrient needs [60]. Future research should examine the relationship between protein intakes and falls and fractures among individuals with diabetes. This would contribute to the understanding of associations between nutrition and common consequences of an aging, at-risk group of adults.

## 5. Conclusions

Diet quality was poorer and physical limitations were more prevalent in adults who consumed less than the recommended protein intakes of 0.8 g/kg/day. Adults with diabetes had more physical limitations than those with non-diabetes, and adults with diabetes who consumed less protein than recommended had further increased limitations. Considering previous studies demonstrate that adults with diabetes are at an increased risk for muscle loss, adequate protein intake may be a critical nutritional factor to help prevent functional decline and sarcopenia. Therefore, our data support the need for nutrition therapy and emphasis on ensuring protein intakes in the adults with diabetes to support healthy aging and reduce or prevent sarcopenia.

## Figures and Tables

**Table 1:** Personal and demographic characteristics in US adults by diabetes status, stratified by intakes above or below protein intake recommendations from the dietary recall.

		Non-Diabetes		Pre-Diabetes		Diabetes	
		Protein Intake ^a^		Protein Intake ^a^		Protein Intake ^a^	
Characteristic	Category	<0.8 g/kg/day (*n* = 5283)	≥0.8 g/kg/day (*n* = 9447)	<0.8 g/kg/day (*n* = 2749)	≥0.8 g/kg/day (*n* = 3120)	<0.8 g/kg/day (*n* = 1482)	≥0.8 g/kg/day (*n* = 1406)
		*n* (%)					
Total		5283(34.2%)	9447 (65.8%)	2749 (45.4%)	3120 (54.6%)	1482 (51.2%)	1406 (48.8%)
Gender	Male	2077 (27.2%)	5033 (72.8%)	1158 (38.4%)	1693 (61.6%)	679 (42.9%)	845 (57.1%)
	Female	3206 (40.4%)	4414 (59.6%)	1591 (51.5%)	1427 (48.5%)	803 (60.7%)	561 (39.3%)
Race/Ethnicity	Mexican American	668 (30.4%)	1456 (69.6%)	346 (38.2%)	537 (61.8%)	273 (42.5%)	343 (57.5%)
	Other Hispanic	472 (32.6%)	884 (67.4%)	266 (43.6%)	324 (56.4%)	138 (40.1%)	176 (59.9%)
	Non-Hispanic White	2707 (34.0%)	4829 (66.0%)	1088 (45.4%)	1217 (54.6%)	499 (51.3%)	443 (48.7%)
	Non-Hispanic Black	1111 (46.5%)	1320 (53.5%)	862 (54.1%)	718 (45.9%)	471 (62.7%)	297 (37.3%)
	Other or Multiracial	325 (25.7%)	958 (74.3%)	187 (37.1%)	324 (62.9%)	101 (48.7%)	147 (51.3%)
Marital Status	Single/Divorced/Widowed	2043 (39.2%)	3002 (60.8%)	1167 (47.8%)	1159 (52.2%)	604 (54.0%)	519 (46.0%)
	Married/Living as Married	3239 (31.9%)	6442 (68.1%)	1581 (44.1%)	1961 (55.9%)	877 (49.8%)	885 (50.2%)
Highest Education Completion	<9th grade	540 (37.8%)	867 (62.2%)	434 (49.3%)	436 (50.7%)	295 (50.6%)	284 (49.4%)
	9–11th grade	763 (38.0%)	1195 (62.0%)	452 (47.3%)	471 (52.7%)	273 (53.6%)	234 (46.4%)
	HS/GED	1247 (38.4%)	1965 (61.6%)	694 (47.5%)	756 (52.5%)	336 (51.9%)	319 (48.1%)
	Some college/Associate’s degree	1600 (38.2%)	2526 (61.8%)	702 (45.9%)	827 (54.1%)	405 (53.8%)	343 (46.2%)
	College grad	1129 (26.4%)	2890 (73.6%)	464 (40.1%)	626 (59.9%)	172 (44.2%)	223 (55.8%)
Meal consumed (%)	Breakfast	4357 (32.5%)	8451 (67.5%)	2343 (43.8%)	2866 (56.2%)	1284 (50.3%)	1284 (49.7%)
	Lunch	3619 (30.8%)	7526 (69.2%)	1854 (42.4%)	2443 (57.6%)	999 (49.1%)	1084 (50.9%)
	Dinner	4576 (32.8%)	8781 (67.2%)	2421 (44.7%)	2854 (55.3%)	1295 (50.6%)	1295 (49.4%)
	Snack	4950 (33.9%)	8992 (66.1%)	2540 (45.2%)	2917 (54.8%)	1360 (50.7%)	1324 (49.3%)
Number of meals reported	1 meal	533 (58.3%)	357 (41.7%)	251 (69.9%)	93 (30.1%)	115 (76.3%)	30 (23.7%)
	2 meals	2117 (44.7%)	2827 (55.3%)	1085 (52.9%)	1005 (47.1%)	605 (53.8%)	495 (46.2%)
	3 meals	2595 (27.9%)	6249 (72.1%)	1399 (40.2%)	2020 (59.8%)	751 (48.0%)	881 (52.0%)
		Mean (95% CI)					
Age (years)		51.9 (51.3, 52.5)	49.8 (49.2, 50.3)	60.1 (59.3, 61.0)	59.1 (58.4, 59.8)	59.8 (59, 60.6)	59 (57.9, 60.2)
Poverty-Income Ratio ^b^		3.0 (2.9, 3.1)	3.4 (3.3, 3.5)	2.7 (2.5, 2.8)	3.0 (2.9, 3.1)	2.6 (2.4, 2.7)	2.8 (2.6, 2.9)
Body Mass Index (kg/m^2^)		30.5 (30.3, 30.8)	27.0 (26.9, 27.2)	33.3 (32.9, 33.8)	29.0 (28.7, 29.3)	36.0 (35.4, 36.6)	30.9 (30.5, 31.3)
Weight (kg)		86.4 (85.6, 87.2)	77.9 (77.4, 78.5)	92.7 (91.4, 94)	81.4 (80.4, 82.3)	100.6 (98.3, 102.9)	88.0 (86.6, 89.5)

^a^ Protein intake recommendations computed as either meeting or not meeting 0.8 g of dietary protein per kilogram of body weight per day. ^b^ Income reported as a ratio of the federal poverty rate.

**Table 2:** Differences in energy and energy-adjusted nutrient intakes across diabetes status for those who were below or met the protein intake recommendation from the 2005–2016 National Health and Nutrition Examination Survey (NHANES) ^a,c^.

	Non-Diabetes			Pre-Diabetes			Diabetes		
	Protein Intake ^a,c^			Protein Intake ^a,c^			Protein Intake ^a,c^		
Nutrients ^b^	<0.8 g/kg/day (*n* = 5283)	≥0.8 g/kg/day (*n* = 9447)	*p*	<0.8 g/kg/day (*n* = 2749)	≥0.8 g/kg/day (*n* = 3120)	*p*	<0.8 g/kg/day (*n* = 1482)	≥0.8 g/kg/day (*n* = 1406)	*p*
Energy (kcal)	1609 (12)	2444 (15)	<0.001	1640 (19)	2318 (21)	<0.001	1551 (25)	2391 (39)	<0.001
Protein, total (g)	52.1 (0.5)	100.2 (0.6)	<0.001	54.4 (0.7)	98.2 (0.8)	<0.001	56.2 (0.9)	104 (1.8)	<0.001
Energy-adjusted intakes									
Protein (g)	33.7 (0.2)	42.8 (0.2)	<0.001	34.7 (0.3)	44.3 (0.3)	<0.001	38.1 (0.5)	45.6 (0.5)	<0.001
Carbohydrate (g)	129 (0.8)	116 (0.5)	<0.001	130 (0.9)	117 (0.8)	<0.001	126 (0.9)	112 (0.9)	<0.001
Added sugars (g)	37.4 (0.6)	28.9 (0.4)	<0.001	36.3 (0.8)	28.2 (0.7)	<0.001	30.5 (1)	22.0 (0.7)	<0.001
Dietary fiber (g)	8.5 (0.1)	8.4 (0.1)	0.526	8.6 (0.1)	8.7 (0.1)	0.569	8.9 (0.2)	9.0 (0.3)	0.732
Total fat (g)	35.8 (0.2)	37.9 (0.2)	<0.001	36.7 (0.3)	39 (0.3)	<0.001	37.9 (0.4)	41.2 (0.4)	<0.001
Saturated fat (g)	11.4 (0.1)	12.2 (0.1)	<0.001	11.8 (0.1)	12.7 (0.1)	<0.001	12.2 (0.2)	13.3 (0.2)	<0.001
Monounsaturated fat (g)	12.8 (0.1)	13.7 (0.1)	<0.001	13 (0.1)	14.1 (0.1)	<0.001	13.5 (0.2)	14.9 (0.2)	<0.001
Polyunsaturated fat (g)	8.5 (0.1)	8.6 (0.1)	0.160	8.6 (0.1)	8.7 (0.1)	0.489	8.7 (0.1)	9.3 (0.1)	0.002
Vitamin A, RAE (µg)	313 (9.0)	323 (5.0)	0.274	297 (8.0)	363 (20.0)	0.002	345 (14.0)	358 (15.0)	0.484
Folate (µg DFE)	263 (4.0)	255 (2.0)	0.046	262 (5.0)	261 (4.0)	0.766	279 (8.0)	270 (7.0)	0.285
Choline (mg)	147 (2.0)	171 (1.0)	<0.001	150 (2.0)	181 (2.0)	<0.001	163 (3.0)	187 (3.0)	<0.001
Vitamin B_12_ (µg)	2.3 (0.1)	2.6 (0.0)	<0.001	2.2 (0.1)	2.8 (0.2)	<0.001	2.5 (0.1)	3.0 (0.1)	0.007
Vitamin C (mg)	48.5 (1.4)	42.2 (0.7)	<0.001	45.2 (1.2)	43.0 (1.2)	0.180	48.0 (2.0)	38.8 (1.3)	<0.001
Vitamin D (µg)	2.0 (0.1)	2.5 (0.1)	<0.001	1.9 (0.1)	2.6 (0.1)	<0.001	2.4 (0.2)	2.7 (0.1)	0.134
Vitamin K (µg)	62.8 (2.4)	61.8 (2.0)	0.730	56.8 (2.7)	64.5 (2.5)	0.028	62.2 (4.3)	65 (4.2)	0.588
Calcium (mg)	440 (5.0)	463 (4.0)	<0.001	433 (5.0)	462 (6.0)	<0.001	496 (14.0)	490 (7.0)	0.697
Phosphorus (mg)	599 (4.0)	683 (3.0)	<0.001	598 (4.0)	696 (5.0)	<0.001	655 (8.0)	734 (8.0)	<0.001
Magnesium (mg)	151 (1.0)	154 (1.0)	0.037	146 (2.0)	151 (1.0)	0.003	150 (3.0)	154 (2.0)	0.205
Iron (mg)	7.2 (0.1)	7.2 (0.1)	0.736	7.3 (0.1)	7.6 (0.1)	0.168	7.9 (0.2)	7.9 (0.2)	0.954
Zinc (mg)	4.9 (0.1)	5.8 (0.01)	<0.001	5.1 (0.1)	6 (0.1)	<0.001	5.5 (0.1)	6.7 (0.3)	<0.001
Copper (mg)	0.65 (0.01)	0.66 (0.01)	0.057	0.64 (0.01)	0.68 (0.03)	0.205	0.66 (0.01)	0.7 (0.02)	0.061
Sodium (mg)	1617 (12.0)	1691 (10.0)	<0.001	1665 (17.0)	1770 (14.0)	<0.001	1764 (23.0)	1868 (28.0)	0.002
Potassium (mg)	1330 (12.0)	1348 (8.0)	0.211	1327 (15.0)	1384 (11.0)	0.002	1399 (30.0)	1407 (18.0)	0.795
Selenium (µg)	47.7 (0.4)	57.9 (0.3)	<0.001	49.7 (0.6)	60.1 (0.5)	<0.001	54.2 (0.8)	62.5 (0.8)	<0.001

^a^ Mean (SE) intakes adjusted for race/ethnicity, gender, marital status and percent of federal poverty rate. ^b^ Nutrients presented as energy-adjusted intakes per 1000 kcal. ^c^ Protein intake recommendations computed as either meeting or not meeting 0.8 g of dietary protein per kilogram of body weight per day.

**Table 3:** Proportion consuming less than the Recommended Intakes across diabetes categories and not meeting the protein recommendation.

	Non-Diabetes		Pre-Diabetes		Diabetes	
	Protein Intake ^b^		Protein Intake ^b^		Protein Intake ^b^	
Nutrient ^a^	<0.8 g/kg/day (*n* = 5283)	≥0.8 g/kg/day (*n* = 9447)	<0.8 g/kg/day (*n* = 2749)	≥0.8 g/kg/day (*n* = 3120)	<0.8 g/kg/day (*n* = 1482)	≥0.8 g/kg/day (*n* = 1406)
Carbohydrate	589 (11.1%)	208 (2.6%)	285 (9.6%)	74 (2.1%)	217 (13.4%)	36 (2.7%)
Fiber	5006 (94.2%)	7720 (81.3%)	2579 (93.4%)	2489 (79.2%)	1378 (93.1%)	1122 (82.1%)
Thiamin	2299 (41.4%)	921 (9.1%)	1123 (36.2%)	315 (9.7%)	569 (33.5%)	118 (7.1%)
Riboflavin	1487 (23.1%)	336 (2.6%)	757 (21.8%)	121 (2.2%)	383 (20.2%)	50 (3.1%)
Niacin	1564 (26.1%)	193 (1.9%)	775 (24.3%)	70 (2.1%)	397 (21.2%)	23 (1.8%)
Vitamin B_6_	2847 (51.1%)	1075 (10.4%)	1549 (52.5%)	486 (14.6%)	869 (54.2%)	187 (12.3%)
Folate	2614 (47.0%)	1727 (18.1%)	1322 (45.8%)	600 (18.0%)	721 (46.0%)	248 (17.1%)
Vitamin B_12_	2291 (40.9%)	1044 (10.0%)	1139 (37.8%)	357 (11.0%)	577 (35.6%)	172 (9.4%)
Vitamin C	3411 (65.4%)	4807 (50.1%)	1722 (64.6%)	1580 (50.7%)	986 (65.7%)	739 (53.3%)
Choline	5144 (97.5%)	7034 (75.1%)	2655 (95.8%)	2358 (76.6%)	1431 (96.0%)	1009 (71.7%)
Vitamin A	3863 (70.8%)	4600 (44.8%)	1958 (68.9%)	1523 (43.2%)	1098 (68.7%)	725 (46.3%)
Vitamin D	4324 (96.8%)	6776 (85.7%)	2398 (97.4%)	2397 (84.2%)	1278 (95.1%)	1072 (85.8%)
Vitamin E	4920 (92.0%)	7211 (73.5%)	2576 (92.9%)	2499 (77.6%)	1393 (91.5%)	1126 (75.3%)
Vitamin K	4284 (78.1%)	6137 (61.6%)	2197 (78.7%)	2030 (61.9%)	1205 (79.0%)	928 (62.8%)
Calcium	4108 (74.5%)	3818 (36.7%)	2179 (77.5%)	1501 (45.5%)	1163 (75.9%)	657 (40.6%)
Phosphorus	1116 (18.4%)	32 (0.3%)	550 (17.0%)	10 (0.2%)	275 (14.2%)	3 (0.1%)
Magnesium	4459 (82.6%)	4091 (40.1%)	2336 (82.2%)	1496 (46.3%)	1285 (84.7%)	681 (46.3%)
Iron	1313 (23.8%)	300 (3.1%)	487 (14.7%)	68 (1.9%)	255 (15.3%)	21 (1.2%)
Zinc	3530 (62.8%)	1453 (13.0%)	1732 (58.2%)	566 (15.8%)	942 (57.7%)	209 (12.3%)
Copper	1944 (33.1%)	561 (4.9%)	926 (31.5%)	236 (6.5%)	505 (30.4%)	85 (4.4%)
Selenium	1259 (22.7%)	66 (0.7%)	558 (17.2%)	11 (0.4%)	277 (15.4%)	2 (0.6%)

^a^ Number; n (% of total). For nutrients without an Estimated Average Requirement, the Adequate Intake is used, italicized above. ^b^ Protein intake recommendations computed as either meeting or not meeting 0.8 g of dietary protein per kilogram of body weight per day.

**Table 4:** Differences in Healthy Eating Index 2015 scores across diabetes status and in those below or meeting the protein intake recommendation.

	Non-Diabetes			Pre-Diabetes			Diabetes		
	Protein Intake ^a,b^			Protein Intake ^a,b^			Protein Intake ^a,b^		
HEI-2015 Score (Score Range)	<0.8 g/kg/day (*n* = 5283)	≥0.8 g/kg/day (*n* = 9447)	*p*	<0.8 g/kg/day (*n* = 2749)	≥0.8 g/kg/day (*n* = 3120)	*p*	<0.8 g/kg/day (*n* = 1482)	≥0.8 g/kg/day (*n* = 1406)	*p*
Total Fruit (0–5)	2.1 (0.05)	2.2 (0.03)	0.174	2.3 (0.07)	2.3 (0.06)	0.664	2.2 (0.09)	2.1 (0.08)	0.565
Whole Fruit (0–5)	2.0 (0.1)	2.2 (0.01)	<0.001	2.2 (0.1)	2.4 (0.1)	0.033	2.2 (0.1)	2.3 (0.1)	0.661
Total Vegetables (0–5)	3.0 (0.01)	3.2 (0.01)	<0.001	3.1 (0.01)	3.2 (0.01)	0.009	3.1 (0.1)	3.4 (0.1)	<0.001
Greens & Beans (0–5)	1.4 (0.01)	1.8 (0.01)	<0.001	1.3 (0.1)	1.8 (0.1)	<0.001	1.2 (0.1)	1.7 (0.1)	<0.001
Whole Grains (0–10)	2.5 (0.1)	2.6 (0.1)	0.533	2.7 (0.1)	2.7 (0.1)	0.995	2.7 (0.1)	2.9 (0.2)	0.471
Dairy (0–10)	4.2 (0.1)	5.2 (0.1)	<0.001	4.3 (0.1)	5.1 (0.1)	<0.001	4.6 (0.1)	5.5 (0.1)	<0.001
Protein Foods (0–5)	3.8 (0.01)	4.5 (0.01)	<0.001	3.9 (0.01)	4.6 (0.01)	<0.001	4.1 (0.1)	4.6 (0.01)	<0.001
Seafood & Plant Proteins (0–5)	2.1 (0.1)	2.7 (0.01)	<0.001	2.1 (0.1)	2.6 (0.1)	<0.001	1.9 (0.1)	2.6 (0.1)	<0.001
Fatty Acids (0–10)	5.4 (0.1)	5.1 (0.1)	0.001	5.2 (0.1)	5.0 (0.1)	0.339	5.3 (0.1)	5.0 (0.1)	0.299
Refined Grains (0–10) ^c^	6.2 (0.1)	6.5 (0.1)	0.014	5.9 (0.1)	6.4 (0.1)	0.002	5.6 (0.1)	6.0 (0.2)	0.123
Sodium (0–10) ^c^	5.0 (0.1)	4.4 (0.1)	<0.001	4.6 (0.1)	3.8 (0.1)	<0.001	4.0 (0.1)	3.2 (0.1)	<0.001
Added Sugars (0–10) ^c^	5.9 (0.1)	7.1 (0.1)	<0.001	6.1 (0.1)	7.2 (0.1)	<0.001	6.9 (0.1)	8.1 (0.1)	<0.001
Saturated Fat (0–10) ^c^	6.6 (0.1)	6.0 (0.1)	<0.001	6.3 (0.1)	5.7 (0.1)	<0.001	6.0 (0.1)	5.2 (0.1)	<0.001
Total HEI Score (0–100)	50.4 (0.3)	53.5 (0.2)	< 0.001	49.9 (0.4)	52.8 (0.4)	<0.001	49.9 (0.5)	52.6 (0.5)	<0.001

^a^ Mean (SE) intakes adjusted for race/ethnicity, gender, marital status and percent of federal poverty rate. ^b^ Protein intake recommendations computed as either meeting or not meeting 0.8 g of dietary protein per kilogram of body weight per day. ^c^ Higher scores for scales related to moderation represent lower intakes.

**Table 5:** Frequency of limitations and grip strength differences across diabetes status in those below or meeting the protein intake recommendation.

	Non-Diabetes		Pre-Diabetes		Diabetes	
	Protein Intake ^a^		Protein Intake ^a^		Protein Intake ^a^	
Limitations Experienced ^b^	<0.8 g/kg/day (*n* = 5283)	≥0.8 g/kg/day (*n* = 9447)	<0.8 g/kg/day (*n* = 2749)	≥0.8 g/kg/day (*n* = 3120)	<0.8 g/kg/day (*n* = 1482)	≥0.8 g/kg/day (*n* = 1406)
	n (%)					
Stooping, crouching, kneeling	1520 (29.0%)	1603 (17.0%)	1104 (40.8%)	930 (30.1%)	760 (52.0%)	488 (35.3%)
Standing for long periods	1346 (25.8%)	1440 (15.4%)	968 (35.9%)	817 (26.5%)	676 (46.9%)	422 (30.6%)
Push or pull large objects	1120 (21.7%)	1216 (13.0%)	784 (29.7%)	695 (22.8%)	570 (40.5%)	343 (25.2%)
Lifting or carrying	793 (15.1%)	815 (8.7%)	587 (21.7%)	484 (15.7%)	447 (30.6%)	267 (19.3%)
Standing up from armless chair	763 (14.5%)	750 (7.9%)	581 (21.2%)	436 (14.0%)	468 (31.6%)	267 (19.0%)
House chores	750 (14.4%)	782 (8.4%)	556 (20.9%)	416 (13.7%)	451 (32.0%)	230 (16.9%)
Sitting for long periods	739 (14.0%)	893 (9.5%)	536 (19.5%)	418 (13.4%)	412 (28.0%)	241 (17.2%)
Going out to movies, events	637 (12.3%)	677 (7.2%)	468 (17.6%)	318 (10.4%)	396 (27.8%)	206 (15.1%)
Walking for a quarter mile	576 (12.3%)	584 (6.6%)	461 (20.5%)	336 (12.2%)	274 (25.7%)	187 (15.8%)
Reaching up over head	628 (11.9%)	644 (6.8%)	448 (16.3%)	354 (11.4%)	358 (24.3%)	238 (17.0%)
Getting in and out of bed	601 (11.4%)	609 (6.4%)	419 (15.3%)	318 (10.2%)	385 (26.1%)	203 (14.5%)
Attending social event	500 (9.8%)	549 (5.9%)	361 (13.8%)	253 (8.4%)	290 (20.9%)	154 (11.5%)
Grasp/holding small objects	500 (9.5%)	647 (6.9%)	366 (13.3%)	319 (10.2%)	296 (20.0%)	181 (12.9%)
Walking up ten steps	422 (9.0%)	393 (4.4%)	312 (13.8%)	235 (8.5%)	204 (19.0%)	133 (11.2%)
Dressing yourself	424 (8.0%)	442 (4.7%)	326 (11.9%)	245 (7.9%)	304 (20.6%)	179 (12.7%)
Walking between rooms (same floor)	314 (5.9%)	307 (3.3%)	270 (9.8%)	174 (5.6%)	240 (16.2%)	120 (8.6%)
Preparing meals	296 (5.7%)	324 (3.5%)	230 (8.7%)	159 (5.3%)	201 (14.4%)	99 (7.3%)
Leisure activity at home	243 (4.6%)	261 (2.8%)	164 (6.0%)	119 (3.8%)	150 (10.2%)	98 (7.0%)
Using fork, knife, drinking from cup	165 (3.1%)	221 (2.3%)	135 (4.9%)	94 (3.0%)	109 (7.4%)	70 (5.0%)
	Mean (SE)					
Number of Limitations (unadjusted)	2.0 (0.1)	1.2 (0.05)	3.1 (0.1)	2.1 (0.1)	4.5 (0.2)	2.7 (0.2)
Number of Limitations (adjusted)	1.7 (0.05)	1.3 (0.05) *	2.8 (0.1)	2.3 (0.1) *	4.3 (0.2)	2.9 (0.2) *
Combined Grip Strength (kg, unadjusted)	70.3 (0.9)	74.1 (0.5) *	67.8 (1.5)	69.6 (1.0)	66.0 (1.8)	70.0 (1.5)
Combined Grip Strength (kg, adjusted)	73.8 (0.5)	72.6 (0.4) *	70.9 (0.9)	68.0 (0.5)	68.0 (0.9)	64.6 (0.8)

^a^ Protein intake recommendation computed as 0.8 g of dietary protein per kilogram of body weight. ^b^ Mean adjusted for race/ethnicity, gender, marital status and percent of federal poverty rate. * Significantly (*p* < 0.001) different between those who met and did not meet the protein intake recommendation within the diabetes category.

## Data Availability

Public use data are available from National Center for Health Statistics of the Centers for Disease Control and Prevention.
